# Ablative Carbon Dioxide Laser as a Therapeutic Option for Multinucleated Cell Angiohistiocytoma

**DOI:** 10.7759/cureus.85361

**Published:** 2025-06-04

**Authors:** David Caetano, Mariana Pedroso, Duarte Flor, José P Reis, Jose C Cardoso

**Affiliations:** 1 Dermatology, Unidade Local de Saúde de Coimbra, Coimbra, PRT

**Keywords:** ablative carbon dioxide, case report, co2 fractional laser, multinucleate cell angiohistiocytoma, multinucleated cells, vascular proliferation

## Abstract

Multinucleated cell angiohistiocytoma (MCAH) is a rare and benign cutaneous proliferation of vascular and histiocytic elements, predominantly affecting the dorsal aspects of acral regions. Clinically, it presents as multiple, flat-topped, violaceous papules that are typically asymptomatic and unilateral, although bilateral cases have been reported. Dermoscopic examination often reveals a fine, whitish reticulated pattern within a violaceous background. Histopathologically, MCAH is characterized by a proliferation of small dermal vessels accompanied by multinucleated cells within a sclerotic stroma. The etiology remains uncertain, but it is generally considered a reactive process. Various treatment modalities have been previously reported with variable success. We report a case of bilateral MCAH in a 56-year-old man, successfully treated with an ablative carbon dioxide laser, resulting in significant lesion reduction and high patient satisfaction.

## Introduction

Multinucleated cell angiohistiocytoma (MCAH) is a rare, benign, histiocytic, and vascular proliferation that mainly affects the dorsal aspect of acral regions, with less than 150 cases reported. It is composed of a proliferation of small vessels in the papillary dermis that clinically presents as multiple 2-15 mm flat-topped and grouped violaceous papules that have a slowly progressive course. These lesions are typically asymptomatic and unilateral, even though bilateral presentations have been described [1]. Dermoscopy often reveals a fine, whitish, reticulated pattern within violaceous papules, corresponding to the vascular components [2]. While the exact etiology and pathogenesis remain uncertain, it is generally considered a reactive process [3]. The differential diagnoses include Kaposi’s sarcoma, lichen planus, granuloma annulare, angiofibroma, and dermatofibroma [4].

Even though spontaneous regression can rarely occur, patients often seek resolution of the lesions, especially for aesthetic concerns [3]. Multiple therapeutic options have been employed, with variable results [5]. To our knowledge, only three cases have reported the use of ablative carbon dioxide laser in MCAH.

## Case presentation

A 56-year-old man presented with a 10-year history of multiple asymptomatic, grouped lesions on the dorsal aspects of both hands. These had been previously interpreted as flat warts and had been unsuccessfully treated with cryotherapy. He reported no history of trauma, chemical exposure, or surgical procedures to the affected areas. He had a past medical history of dyslipidemia and type 2 diabetes, for which he took simvastatin and metformin daily.

**Figure 1:**
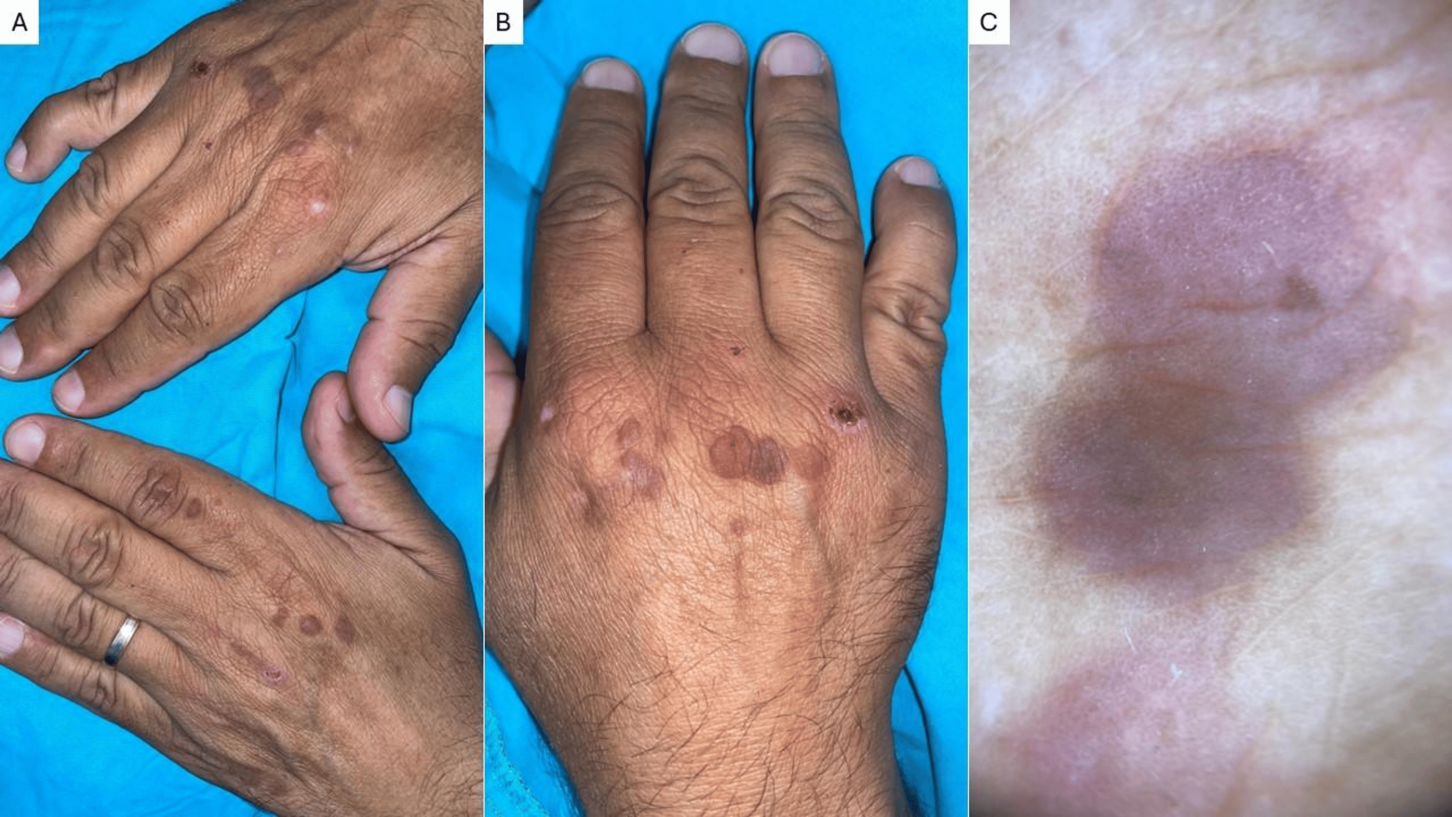
Clinical and dermoscopical photographs of multinucleated cell angiohistiocytoma Multiple violaceous, flat-topped papules on the dorsal aspects of both hands (A), some of which are grouped and coalesce into plaques (B). Dermoscopy shows diffuse violaceous areas with a whitish fine reticulated pattern (C). Note a traumatic abrasion over the right fourth metacarpophalangeal joint.

Examination revealed multiple, well-defined, firm, violaceous papules (5-10 mm) on the dorsal hands, some coalescing into small plaques with a mildly papillomatous surface (Figure 1A, 1B). Dermoscopy revealed scattered violaceous areas with a fine reticulated pattern in the center, without visible and individualized vessels or other structures (Figure 1C).

Histopathological examination revealed a mildly acanthotic and papillomatous epidermis, with proliferation of slightly thickened capillaries in the dermis, surrounded by a sclerotic stroma containing multiple multinucleated cells, some exhibiting dendritic morphology. The clinical and histological findings were those of multinucleated cell angiohistiocytoma.

**Figure 2:**
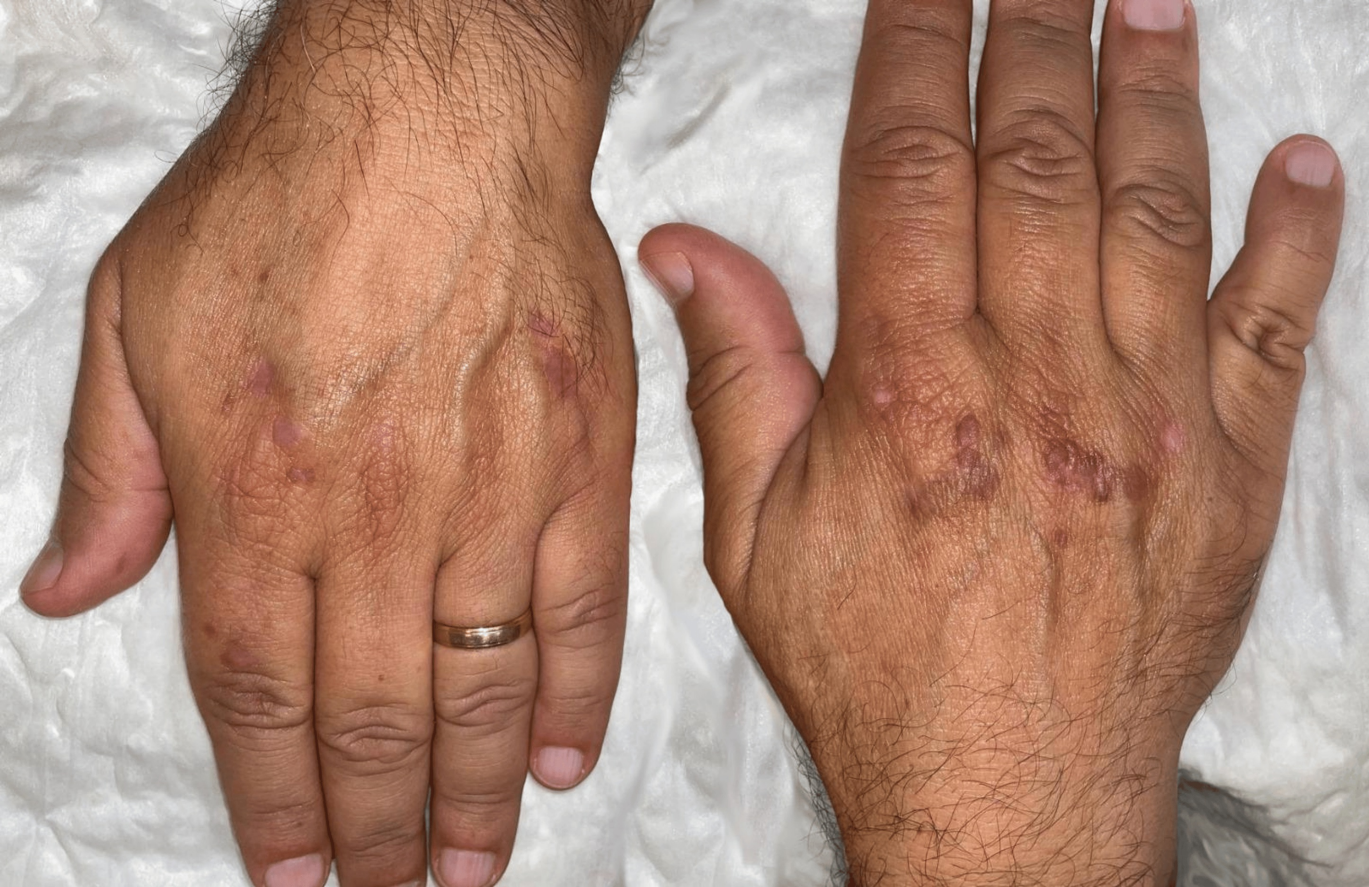
Partial resolution of the left-hand lesions after ablative carbon dioxide laser Partial clearing of lesions on the dorsal aspect of the left hand after the first session of ablative carbon dioxide laser, contrasting with the yet untreated lesions on the dorsal aspect of the right hand

Due to the patient's aesthetic concerns and desire for removal, the lesions were treated under local anesthesia (2% lidocaine) using an ablative carbon dioxide laser in a continuous wave mode, focused mode, with a power density of 2 to 3 W/cm², adjusted according to lesion depth. The procedure was initially performed on the left hand, leading to a significant reduction in both the number and size of lesions, with minimal scarring and high patient satisfaction after one month (Figure 2). Given the favorable outcome, the same procedure was subsequently performed on the right hand, yielding comparable results. No recurrences were observed after a six-month follow-up.

## Discussion

This case highlights the importance of recognizing this dermatological entity, especially regarding the differential diagnosis of violaceous cutaneous lesions presenting in acral areas, and presents a therapeutic option for patients who seek the resolution of the lesions.

MCAH is a rare benign proliferation of histiocytic and vascular origin. The lesions are usually asymptomatic and unilateral, with bilateral presentations being rare, and patients often report only aesthetic concerns, which motivate them to seek medical care. To date, no associations with malignancy or systemic conditions have been reported. A histopathological analysis is key for establishing the diagnosis by showing a proliferation of small vessels in the dermis with adjacent multinucleated cells within a sclerotic stroma [1,3].

Although the course is benign and there are scattered reports of spontaneous regression, progression is usually slow and persistent. Given its benign nature, an initial conservative approach may be recommended. However, for patients seeking resolution, an ablative carbon dioxide laser appears to be an effective therapeutic option, offering satisfactory results with minimal scarring. Surgical excision, repeated sessions of cryotherapy, argon laser, and pulsed dye laser are some of the alternatives that are reported in the literature, with variable success [3,5].

## Conclusions

MCAH is an uncommon, benign, vascular, and histiocytic proliferation with variable clinical presentations. Histopathological analysis remains the cornerstone for diagnosis. While the condition is benign and may not require treatment, aesthetic concerns often prompt intervention. Ablative carbon dioxide laser therapy appears to be an effective treatment modality, offering satisfactory cosmetic outcomes with minimal adverse effects. Further studies are needed to evaluate its long-term outcomes and recurrence rates.
